# Supplementary material for: Expression Patterns of the Heat Shock Protein 90 (Hsp90) Gene Suggest Its Possible Involvement in Maintaining the Dormancy of Dinoflagellate Resting Cysts
Source: Int J Mol Sci. 2021 Oct 13;22(20):11054. doi: 10.3390/ijms222011054 (PMC8538777; doi:10.3390/ijms222011054)
Supplement: Supplementary file 1 [file ijms-22-11054-s001.zip › Supplementary Figures.pdf]

Supplementary Figures

1 TCCGTAGCCATTTTGGCTCAAGTTTTGGCTCAAGCAACTTACATCAAGCTCGTGCTGTAGATTGCCTCGTCCTTGCCTCTGCCAGCATTGCCGGAGAGTCT

102 GCGCAATGGCGGAGACATTTGCTTTCAATGCGGACATCCAGCAGCTGATGAGCTTGATCATCAACACCTTCTACTCCAACAAGGAGATCTTCCTCCGAGA

1 M A E T F A F N A D I Q Q L M S L I I N T F Y S N K E I F L R E

202 GCTCATCTCCAATGCCTCAGATGCCTTGGACAAGATCCGCTACGAGTCCATCACGGACCCGGAGAAGATTGAGGCGCAGCCCAACTTCTACATCAAGATC

33 L I S N A S D A L D K I R Y E S I T D P E K I E A Q P N F Y I K I

302 ATCCCTGACAAGACCAACTCCACCATCACCATCGAGGACTCTGGCATCGGCATGACGAAGAATGAGCTGATCAACAACCTCGGCACCATCGCCAAGTCC

66 I P D K T N S T I T I E D S G I G M T K N E L I N N L G T I A K S

401 GGCACCAAGGCCTTCATGGAGGCCATGGCAGCGGGCGGCGACATCTCCATGATCGGCCAGTTCGGCGTGGGCTTCTACTCCGCCTACCTCGTGGCAGAC

99 G T K A F M E A M A A G G D I S M I G Q F G V G F Y S A Y L V A D

500 AAGATCCGCGTTGTGAGCAAGCACAACGACGACGAGCAGTACATCTGGGAGTCGGGGGCTGGCGGCTCCTTCACGGTGCAGAAGGACACGGAGATGGT

132 K I R V V S K H N D D E Q Y I W E S G A G G S F T V Q K D T E M V

598 GCACGGCGAGATCAAGCGCGGCACGAAGATCATCTGCTACCTGAAGGAGGACCAGTCCGAGTTCCTCGAGGAGCGCCGCCTGAAGGACCTGGTGAAGA

165 H G E I K R G T K I I C Y L K E D Q S E F L E E R R L K D L V K

696 AGCATTCGGAATTTATTGGCTTCCCGATCGAGCTCTACGTGGAGAAGTCCAAGGAGAAGGAGGTCACCGACTCGGAAGAGGAGGAGGAGGAGAAGAAG

197 K H S E F I G F P I E L Y V E K S K E K E V T D S E E E E E E K K

794 GAGGAGGGCGCCGAGGGTGACGAGCCGAAGATTGAGGAGGTGGACGAGGAGAAGGAGAAGGAAGAGAAGAAGAAGAAAACCAAGAAGGTCAAGGA

230 E E G A E G D E P K I E E V D E E K E K E E K K K K T K K V K E

889 AGTTTCTCATGAGTGGGAACAGCTGAACAAGAACAAGCCCCTCTGGATGCGGAAGTCGGAGGACGTGACGAATGAGGAGTATGCCTCCTTCTACAAGTC

262 V S H E W E Q L N K N K P L W M R K S E D V T N E E Y A S F Y K S

988 GCTCTCGAATGATTGGGAGGACCATCTTGCCGTGAAGCACTTCAGCGTCGAGGGGCAGCTCGAGTTCCGAGCCTTGCTCTTCGTGCCGCGCCGCGCACCT

295 L S N D W E D H L A V K H F S V E G Q L E F R A L L F V P R R A P

1088 TTCGACCTCTTTGAGTCCAAGAAGAAGAGGAACAACATCAAGTTGTACGTGCGCCGCGTCTTCATTATGGACGATTGCGATGAGCTCATGCCCGAGTGGC

328 F D L F E S K K K R N N I K L Y V R R V F I M D D C D E L M P E W

1188 TCAACATGGTCAAGGGCGTCGTGGATTCGGAGGATCTGCCGCTGAACATCTCTCGAGAGACCTTGCAGCAGAACAAGATCCTCCGCGTCATCAAGAAAAA

361 L N M V K G V V D S E D L P L N I S R E T L Q Q N K I L R V I K K N

1288 CCTTGTGAAGAAGTGTTTGGAGATGTTTGCTGAGATCGCGGAGAAGAAGGATGACTACAAGAAATGTTACGAGCAGTTTGGCAAGTGCTTGAAGCTCGGC

395 L V K K C L E M F A E I A E K K D D Y K K C Y E Q F G K C L K L G

1388 GTCCACGAAGATTCCACCAACCGAACCAAGGTTGCAGAGTTGCTCCGCTTCCACACTTCCAAGTCTGGCGATGAGCAGATCAGCTTGAAGGAGTATGTGG

428 V H E D S T N R T K V A E L L R F H T S K S G D E Q I S L K E Y V

1488 ACCGCATGAAAGAGGGCCAGAACGACATCTACTACATCACTGGCGAGAGCATCACGGCCGTGTCCTCCTCGCCGTTTTTGGAGACCCTTCGCAAGAAGGG

461 D R M K E G Q N D I Y Y I T G E S I T A V S S S P F L E T L R K K G

1588 CTTGGAGGTGTTGTACATGGTGGACCCGGTTGACGAGTATGCGGTGCAGCAGTTGAAGGAGTTCGATGGTAAGAAGCTCAAGTCCACGACCAAGGAGGG

495 L E V L Y M V D P V D E Y A V Q Q L K E F D G K K L K S T T K E G

1687 CTTGGACATTGAGGACGAGGATGAGAAGAAGAAGCTTGAGGAATTGAAGGCCGAGTTTGAGCCACTTACGAAGCTCATGAAGGAGGTTCTTGGCGACAA

528 L D I E D E D E K K K L E E L K A E F E P L T K L M K E V L G D K

1786 GGTGGAGAAGGTCCTCGTGAGCTCGCGCATGGCTGACTCCCCTTGCGTGCTCACGACCTCGGAGTATGGCTGGTCGGCGAACATGGAACGCATCATGAAG

561 V E K V L V S S R M A D S P C V L T T S E Y G W S A N M E R I M K

1886 GCGCAGGCTTTGCGCGACAACTCTATGACGTCGTACATGGTGTCGAAGAAGACCATGGAGGTGAATCCGAAGCACTCCATCATGACGGAGTTGAAGAAGA

594 A Q A L R D N S M T S Y M V S K K T M E V N P K H S I M T E L K K

1986 AGGCTGCCGCGGACAAGTCTGACAAGACTGTGAAGGACCTGATCTGGCTGCTCTTCGACACCTCGCTGCTCACGTCCGGCTTCAACCTGGACGAGCCCAC

627 K A A A D K S D K T V K D L I W L L F D T S L L T S G F N L D E P T

2086 GCAGTTCGCTGGCCGGATCCACCGCATGATCAAGCTCGGCCTCAGCATCGACGATGACGATGAGGGCCTCGGTGACGATGACGACCTTCCCCCGCTCGAA

661 Q F A G R I H R M I K L G L S I D D D D E G L G D D D D L P P L E

2186 GAGGTCGAGGGCGCTGCGGATGAGGCCTCCAAGATGGAGGAGGTCGATTGAGGCGCCCATGCAGTGAGCGACGGCCTCCACGGGCCGCCCGACTTGGG

694 E V E G A A D E A S K M E E V D -

2284 CCGCTGGTGATCAATGAGGCTCAGCACAGTATCACCACGCAGAGCCTCCGCCGACCGCATGGGGAGGAGCGTAGTCGAAGGTGCTACATGCGAGCGCGT

2383 CCGCCCGCTTGGGGCGCGGCGTAGAGACCCAGGACCTTGCTGCATGTACAGTGAGAGAGGCAGGAGCAGAAAAAAAAAAAAAAAAAAAAA

Supplementary Figure S1. The full-length cDNA sequence and deduced amino acid sequence of *StHsp90* (Accession number: MZ779085). Sequences are numbered on the left. The start and stop codon are boxed. The canonical dinoflagellate spliced leader (DinoSL) is underlined in the 5′-UTR. The characteristic Hsp90 signatures are shaded in gray and the conserved LXXLL sequence is marked with double underline. The cytosolic Hsp90-specific motif at C-terminus is highlighted with triangles.

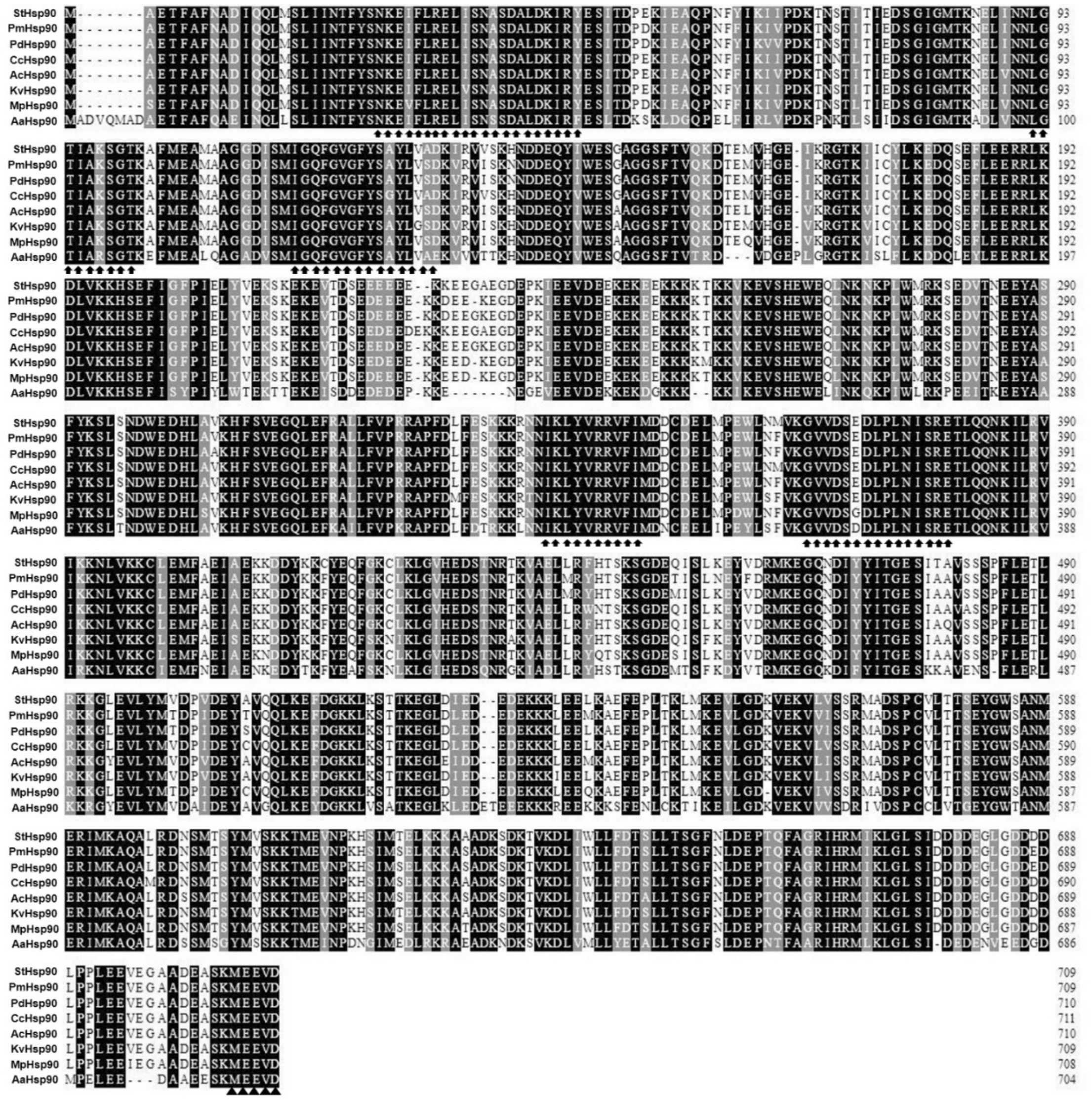

Supplementary Figure S2. Alignment and comparison of Hsp90 deduced amino acid sequences from *Scrippsiella trochoidea* (StHsp90, the present sequence) with other registered counterparts from dinoflagellates *Prorocentrum minimum* (AFD34191; PmHsp90), *Prorocentrum donghaiense* (AST24279; PdHsp90), *Crypthecodinium cohnii* (AAM02974; CcHsp90), *Margalefidinium polykrikoides* (AKS44062; MpHsp90), *Amphidinium carterae* (ADV03069; AcHsp90), Karlodinium veneficum (ABI14419; KvHsp90), and higher plant *Arabidopsis thaliana* (CAA68885; AaHsp90). Sequences are numbered on the right. The typical Hsp90 signatures and the cytosolic Hsp90-specific motif are highlighted with arrows and triangles, respectively. Identical and similar amino acid residues are black and gray shaded, respectively. Deletions are indicated by dashes.

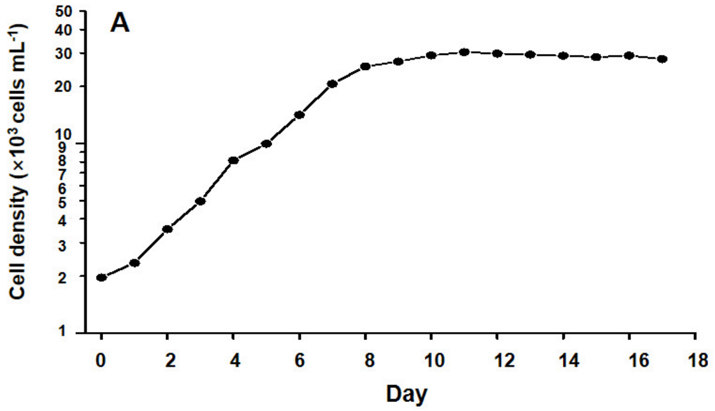

Supplementary Figure S3. The growth curve of *Scrippsiella trochoidea* (strain IOCAS-St-1). The day of inoculation was recorded as day 0.
